# Analytical cohort study on splenomegaly and cytopenias in myeloproliferative disorders

**DOI:** 10.6026/973206300214678

**Published:** 2025-12-15

**Authors:** Sarvodayai Sinha, Vishnu Poovathinkal Rajan, Aditya Unnikrishnan Nair, Achyuth Prasad Jakka, Sandhya Palayapadi Chandrasekaran

**Affiliations:** 1 Department of Critcal Care, Aster Hospital, Muhaisnah, Dubai, UAE; 2 Department of General Medicine, ESIC Hospital, Udyogamandal, Ernakulam, Kerala, India; 3 Department of General Medicine, Altnagelvin Hospital, Western Health and Social Care Trust, Northern Ireland, UK; 4 Department of General Medicine, Government Medical College, Anantapuramu andhra Pradesh, India; 5 Department of General Medicine, Sree Balaji Medical College and Hospital, Chrompet, Chennai, Tamil Nadu, India

**Keywords:** Myeloproliferative diseases, splenomegaly, hematologic parameters, The severity of the condition

## Abstract

The relationship between splenomegaly and cytopenias in myeloproliferative disorders (MPDs) remains poorly defined despite its clinical importance. This analytical cohort study evaluated 124 patients with MPDs, including polycythemia vera, essential thrombocythemia, and primary myelofibrosis, to characterize associations between splenic enlargement and hematologic abnormalities. Splenomegaly was present in 68.5% of patients and showed significant correlations with anemia and thrombocytopenia. Individuals with massive splenomegaly demonstrated greater symptom burden and more pronounced pancytopenia. These findings suggest that splenic size may serve as an important indicator of disease severity and may guide early therapeutic decision-making in MPD management

## Background:

One of the characteristics of a wide range of clonal hematologic malignancies known as myeloproliferative disorders (MPDs) is the proliferation of one or more myeloid cell lines [1]. The classic MPDs include polycythemia Vera (PV), essential thrombocythemia (ET) and primary myelofibrosis (PMF) [2]. Common clinical features of these disorders include constitutional symptoms, elevated blood counts, thrombotic events and most importantly splenomegaly [3]. A hallmark of MPDs, splenomegaly is both diagnostic and prognostic, particularly in myelofibrosis, where increasing spleen enlargement is caused by extramedullary hematopoiesis [4]. As MPDs progress, bone marrow fibrosis, inefficient hematopoiesis and hypersplenism frequently result in cytopenias, including anemia, leukopenia and thrombocytopenia [5]. Increasing transfusion reliance and exacerbating cytopenias can result from an enlarged spleen actively sequestering circulating blood cell [6]. Thrombocytopenia and neutropenia might appear in advanced stages or after treatment, although anemia is more commonly seen in myelofibrosis [7]. Gaining knowledge about the connection between spleen size and hematologic parameters may help one better understand the severity, course and responsiveness to therapy of the disease [8]. Few analytical or prospective investigations, nevertheless, have thoroughly examined this connection across all of the main MPD subtypes [9]. By evaluating the relationship between the degree of splenomegaly and the frequency and severity of cytopenias in MPD patients, this analytical cohort study seeks to inform therapeutic decision-making and clinical monitoring tactics [10]. Therefore, it is of interest to study and describe the association between splenomegaly and cytopenias in myeloproliferative disorders to guide monitoring and treatment strategies.

## Materials and Methods:

This 18-month analytical cohort study was carried out in a tertiary care center's hematology department from January 2023 to June 2024. According to WHO 2016 criteria, 124 adult patients with a diagnosis of primary myelofibrosis (PMF), essential thrombocythemia (ET), or polycythemia vera (PV)-classic myeloproliferative disorders-were enrolled. In order to prevent conflicting cytopenia causes, patients with concomitant infections, autoimmune diseases, ongoing chemotherapy, or other hematologic malignancies were not included. Comprehensive clinical histories were taken at the time of enrollment, covering constitutional symptoms, bleeding patterns and the need for transfusions. The size of the spleen was measured by palpation during physical examinations and abdominal ultrasonography was used to corroborate the results. Splenomegaly was graded as mild (<5 cm below the costal margin), moderate (5-10 cm), or massive (>10 cm). Laboratory investigations included complete blood count (CBC), peripheral smear analysis, serum LDH, bone marrow biopsy and JAK2 V617F mutation status. Cytopenias were defined as hemoglobin <12 g/dL (anemia), platelet count <150 x 10^9^/L (thrombocytopenia) and total leukocyte count <4 x 10^9^/L (leukopenia). The primary objective was to analyze the association between splenic size and prevalence/severity of cytopenias. Secondary objectives included correlation with symptom burden and transfusion needs. Data were analyzed using SPSS version 26. Chi-square tests were used for categorical variables and ANOVA or Kruskal-Wallis tests were applied for continuous variables. Multivariate logistic regression was employed to identify independent predictors of cytopenias. A p-value of <0.05 was considered statistically significant.

## Results:

A total of 124 patients with myeloproliferative disorders (MPDs) were included. The most common diagnosis was Primary Myelofibrosis (PMF), followed by Polycythemia Vera (PV) and Essential Thrombocythemia (ET). Splenomegaly was present in 85 patients (68.5%), with massive splenomegaly observed in 29.8% of cases. Anemia was the most frequent cytopenia, followed by thrombocytopenia and leukopenia. There was a significant association between the severity of splenomegaly and both the presence and extent of cytopenias. Multivariate analysis revealed that massive splenomegaly, PMF diagnosis and elevated LDH were independent predictors of cytopenias.

Table 1 (see PDF) shows that primary myelofibrosis accounted for the largest proportion of cases, followed by polycythemia vera and essential thrombocythemia. Table 2 (see PDF) displays that splenomegaly was present in more than two-thirds of patients, with nearly one-fifth having massive enlargement. Table 3 (see PDF) shows that anemia and thrombocytopenia were significantly more common among patients with splenomegaly. Table 4 (see PDF) displays that primary myelofibrosis patients had the highest rates of both splenomegaly and cytopenias compared to other subtypes. Table 5 (see PDF) shows that patients with massive splenomegaly had the lowest hemoglobin and platelet counts, indicating severe cytopenias. Table 6 (see PDF) displays that LDH levels rose progressively with increasing spleen size, peaking in massive splenomegaly. Table 7 (see PDF) shows that symptom burden scores also increased with spleen enlargement. Table 8 (see PDF) demonstrates that transfusion dependence was highest among patients with massive splenomegaly. Table 9 (see PDF) shows multivariate regression identified massive splenomegaly, primary myelofibrosis and high LDH as independent predictors of cytopenias. Finally, Table 10 (see PDF) displays that cytopenic patients had significantly worse ECOG performance status and delays in therapy initiation compared to non-cytopenic patients.

## Discussion:

This analytical cohort study reinforces the central role of splenomegaly as both a marker of disease burden and a contributor to hematologic complications in myeloproliferative disorders (MPDs). Among the 124 patients, nearly 69% exhibited splenomegaly, with massive splenomegaly present in over 21% [11]. These findings are consistent with known pathophysiologic mechanisms of MPDs, particularly primary myelofibrosis (PMF), where extramedullary hematopoiesis leads to progressive spleen enlargement. Cytopenias especially anemia and thrombocytopenia were significantly more prevalent in patients with splenomegaly; supporting the hypothesis that hypersplenism contributes to peripheral cell sequestration and destruction [12]. The severity of cytopenias closely paralleled the extent of splenic enlargement, suggesting a dose-response relationship. Patients with massive splenomegaly had the lowest hemoglobin and platelet counts and the highest transfusion requirements. Due to the advanced fibrotic marrow and extramedullary hematopoiesis characteristic of PMF, this link was highest in these patients [13]. Patients with larger spleens also had higher serum LDH levels, according to the study, which is indicative of rapid cell turnover and disease activity [10]. Significantly, PMF subtype, increased LDH and significant splenomegaly were found to be independent predictors of cytopenias by multivariate analysis. These results highlight how important spleen size is for prognosis and how useful it is to incorporate splenic evaluation into regular MPD monitoring [14]. Clinically, cytopenic individuals had delayed commencement of disease-modifying treatment, including JAK inhibitors and significantly worse functional status (ECOG ≥2). In patients with severe cytopenias and splenomegaly, prompt management is crucial since this delay may have an impact on long-term survival and quality of life. It is hard to link the results with survival outcomes or the onset of acute leukemia due to the study's single-center design and lack of longitudinal follow-up data [15]. Nevertheless, the evidence clearly demonstrates that splenomegaly is a clinically relevant and quantifiable aspect of MPDs that influences cytopenias, functional outcomes and treatment decisions. Conclusion: Spleen size, particularly high splenomegaly, should be considered a proxy for disease severity and a predictor of cytopenias in MPDs. High-risk individuals may experience fewer consequences if they are identified early and treated with specialized techniques, such as early JAK2 inhibitor initiation or splenectomy consideration. The therapeutic implications of focusing on splenic involvement in MPDs require further prospective research with longitudinal outcomes [16].

## Conclusion:

Cytopenias are substantially linked to splenomegaly, especially in its large form in myeloproliferative diseases. It influences therapy choices and patient outcomes as a potent indicator of the severity of the disease. Thus, spleen size must be regularly measured for risk categorization and early intervention.
